# Correlates of post-COVID-19 pandemic worry and preventive practices in older adults in Florida

**DOI:** 10.3389/fpubh.2025.1608352

**Published:** 2025-07-09

**Authors:** Mirza M. Baig, Lilah M. Besser, Magdalena I. Tolea, Michael J. Kleiman, Lun-Ching Chang, Deirdre M. O’Shea, Stephanie Chrisphonte, Lisa K. Wiese, James E. Galvin

**Affiliations:** ^1^Department of Neurology, Comprehensive Center for Brain Health, University of Miami, Boca Raton, FL, United States; ^2^Department of Mathematics and Statistics, Florida Atlantic University, Boca Raton, FL, United States; ^3^Christine E. Lynn College Nursing, Florida Atlantic University, Boca Raton, FL, United States

**Keywords:** post-COVID-19 pandemic, COVID anxiety, preventive practices, older adults, South Florida

## Abstract

**Introduction:**

The extant literature is limited regarding the correlates of COVID-19 worry and preventive measures among diverse, older adults following the peak of the pandemic. Purpose of the study is to determine the correlates of post-COVID-19 pandemic worry and preventive practices (i.e., social distancing, masking) among older adults.

**Materials and methods:**

In 2022–2024, we conducted a cross-sectional survey of COVID-19 preventive behaviors, concerns, beliefs, and trusted sources of information in older adults in rural and urban/suburban settings in south-central Florida. A convenience sample of 522 English-speaking participants age 60 years or older were recruited using word-of-mouth, flyers, and recruitment events in urban, suburban, and rural settings. Comparisons were made for COVID-19 worry and preventive practices across key sociodemographic variables (e.g., age, sex, race/ethnicity, education, vaccination status, residence, and COVID-19 testing history) using multivariable linear and logistic regression models.

**Results:**

Participants (mean age 72 ± 9 years) were predominantly women (73%), Black (74%), and rural residents (57%). Greater COVID-19 worry was higher among participants who were younger, women, less educated, vaccinated, rural residents, never testing positive for COVID-19, and trusted authorities for health information. Black and Hispanic participants, as well as vaccinated individuals, were more likely to mask in public, while women and Black and Hispanic participants were more likely to practice social distancing.

**Discussion:**

Post-COVID-19 pandemic worry and preventive practices were correlated with demographics, vaccination status, and trust in health authorities. The findings underscore the importance of targeted public health messaging and interventions that consider the distinct needs and concerns of diverse older adult populations. This study’s explicit focus on sociodemographic differences provides valuable insights for designing more effective pandemic preparedness and response strategies tailored to vulnerable and diverse communities.

## Introduction

A highly transmissible and pathogenic contagion has claimed over 16 million deaths globally, and 2.2 million deaths in the United States, as of January 2025. The cause of this pandemic, identified as a strain of acute respiratory syndrome coronavirus 2 (SARS-CoV-2), is commonly referred to as COVID-19 (novel coronavirus 2019). Incidence and mortality rates throughout the COVID-19 pandemic have varied considerably across states (1).

In the early days of the pandemic when effective vaccines were still being developed, public health efforts focused on encouraging preventive behaviors like masking and social distancing. Studies have shown that digital health information-seeking practices and COVID-19 testing status influenced compliance with these preventive measures (2). Demographic and clinical predictors of COVID-19 severity and mortality were identified, including age, gender, preexisting comorbidities and biomarkers (e.g., white cell count and markers of inflammation) (3) and guided public health strategies and clinical management (4).

Over time, preventive practices and beliefs shifted as vaccines became available, new variants emerged, and public health guidelines evolved. However, there is limited literature on the temporal changes related to practices and beliefs associated with individual behaviors in the post-peak pandemic period when vaccines became readily available (5). Furthermore, a significant gap in the literature exists regarding the predictors of post-COVID-19 practices following the peak of the pandemic among older adults from diverse racial, ethnic, and geographic groups. These populations are particularly vulnerable to severe COVID-19 outcomes and may have distinct patterns of preventive behavior adoption.

Focusing on these older adult subgroups in the post vaccine phase is crucial not only because of their vulnerability but also because they may have unique patterns of vaccine uptake, information-seeking, and adherence to preventive measures. Older adults and especially minorities who reside in rural areas are more likely to experience severe outcomes from COVID-19, and their health behaviors are influenced by access to care, social support, and trust in health authorities, which may evolve in the post-vaccine context (6, 7). Yet, few studies have examined these dynamics specifically among older adults after the pandemic’s peak.

To address these gaps, this study investigated the prevalence and correlates of post-COVID-19 worry and preventive practices including vaccination, social distancing, and masking among older adults in rural, urban and suburban regions of south Florida. Our research aimed to uncover valuable information that can be directly applied to tailored public health approaches and mitigate health risks for susceptible populations in future pandemic scenarios.

## Materials and methods

The Comprehensive Center for Brain Health at the University of Miami conducted an NIH-funded study to assess the root causes of health disparities associated with Alzheimer’s disease and related disorders (ADRD), specifically vascular contributions to cognitive impairment and dementia (VCID) in older adults living in rural or urban/suburban settings in South Florida. As an administrative supplement to the VCID study, a brief COVID-19 survey was administered in 2022–2024 in a subset of participants to identify trends relating to COVID-19 exposure, symptoms, preventive practices, attitudes, beliefs, and health literacy. The purpose of the survey was to determine how the pandemic impacted different sociodemographic groups and identify variations in post-COVID-related health practices and preventive measures unique to each group. The survey was gaged to also identify the specific trusted sources of information used by the public.

Participants were recruited through several methods including word-of-mouth, flyers, and announcements by local pastoral and civic leaders in monthly meetings, recruitment events were held in churches and community centers to enroll participants residing in urban, suburban, and rural settings. The survey (Supplementary Table 1) was administered on paper and the data was collected between November 2022 and February 2024. The University of Miami Institutional Review Board (IRB) approved the COVID-19 questionnaire and written informed consent was obtained from the first 114 participants. The participants in rural communities expressed concern that their responses could be linked to their personal information and whether there could be deleterious ramifications for their community which was already underserved and under resourced. After discussion with community leaders and the research team, the protocol was revised to anonymize the responses and a waiver of consent was obtained from the IRB.

The survey instrument was originally designed using scales from an NIH-provided toolkit (8) and included questions on COVID-19 beliefs, behaviors, and social norms (9), self-testing, symptoms, hospitalizations, recovery and reinfection (10), health care, racial discrimination, and the disproportionate burden of COVID-19 on the Black community (11). The NIH grant was awarded at the height of the COVID-19 pandemic which coincided with a pause of in-person research activities at the University of Miami. By the time of restarting research activities, Operation Warp Speed was in full gear and vaccination soon became available and was readily utilized in the Glades (12). These original survey questions contained hypothetical scenarios about health behaviors should a vaccine become available and were therefore no longer relevant. We received feedback from the community and redesigned the survey to capture post-COVID behaviors.

The revised survey was crafted based on community input to emphasize whether respondents were vaccinated and the impact of the pandemic on changes in and sustainability of health behaviors based on personal experience with the pandemic. Additional questions were developed to identify trusted sources of health information from media, local, state, and federal agencies. The revised survey included questions on sociodemographic characteristics, COVID-19 diagnosis and treatment history, vaccination status, preventive practices (e.g., masks, social distancing) and reaction to others who either did or did not use preventive practices, the extent to which they believed the local, state and federal government was acting in their best interest, their view of the credibility of the information they received from authorities and media, and their general worry about COVID-19 now and in the future (Supplementary Table 1).

Socio-demographics included age (in years), sex, race, ethnicity (Hispanic or non-Hispanic), years of education, and geographic locale (Rural or Urban/Suburban). Information on personal history of COVID-19 (Never tested positive vs. ever tested positive for COVID-19), vaccination status, and history of antiviral treatment for COVID-19 was also provided. Participants were asked to report on preventive practices including social distancing and their mask-wearing practices and their attitudes toward other community members who either did or did not perform these preventive practices. As prior data exists suggesting a clear increase in COVID-19 severity and mortality risk for those aged 70 and above (13), age was further dichotomized as <70 years and ≥70 years. Years of education (YOE) was additionally categorized as ≤ 12 years, and >12 years. Based on the federal designations of a rural region (14), the western areas of Palm Beach County, Florida, an economically disadvantaged inland region near the Everglades and Lake Okeechobee, colloquially known as “The Glades” were classified as rural. The more urbanized and generally affluent coastal areas of Palm Beach and Broward County, colloquially referred to as “The Coast” were classified as urban/suburban.

Post-COVID-19 worry was assessed with 12 newly developed questions crafted to gage the unease surrounding the pandemic’s long-term impact on health (psychological and physiological), vaccine-related long-term effects, individual financial situations, government response and regulations, return to pre-pandemic normalcy, and the potential for future disease outbreaks. The questions were scored on a scale of 1–10, with 0 indicating the lowest worry and 10 the highest worry. Two belief factors were measured including (1) the extent to which respondents believed that the various levels of governments (federal, state, county, city) were genuinely acting in the public’s best interests and (2) the credibility of the information disseminated by the government, pharmaceutical companies, doctors, and TV news programs. In total, there were 8 newly developed belief related questions (four for each belief measure), each scored on a 4-point Likert scale from 0: Strongly Disagree, 1: Disagree, 2: Agree, 3: Strongly Agree.

Principal component analysis was then used to reduce the dimensionality of the belief and worry related questions, and three indices were created for every participant: (1) The Best Interest Index, calculated by averaging the responses to questions related to believing that the various levels of governments were acting in the public’s best interests; (2) The Believe Information Index calculated by averaging the responses to questions assessing the participant’s trust in information from various sources such as the government, pharmaceutical companies, doctors, and TV news programs; and (3) The COVID-19 Worry Index, which was calculated by averaging the responses to the 12 questions related to concerns about the pandemic. These indices were used to create categorical variables using the average score for belief indices (0–1: Disagree; 2–3: Agree) and the median value for the worry index (<4.67: Low Worry; ≥4.67: High Worry).

The three indices were constructed specifically for this study and were not adapted from previously validated scales. Item selection was guided by principal component analysis, which identified which questions naturally grouped together into meaningful clusters.

While the indices were not pilot tested due to time and resource constraints, their reliability was assessed with Cronbach’s alpha and the corresponding 95% confidence intervals (CI) calculated for each index. Cronbach’s alpha is a widely used measure of internal consistency, indicating the extent to which the items within each index are related to each other (15).

Descriptive statistics (e.g., means and standard deviations, counts, and percentages) were calculated for the participants’ demographics (e.g., age, education), the three indexes, and COVID-related variables (e.g., vaccination status, previous positive test for COVID-19).

Multivariate linear regression analysis was used to calculate the association between participant characteristics (e.g., belief, age, sex) and the COVID-19 Worry Index. To assess the association between participant characteristics and COVID-19 preventive practices, specifically social distancing and mask-wearing, multivariate logistic regression models were used to calculate adjusted odds ratios (AOR) and 95% CI. The variables included in the multivariate linear and logistic regression models were chosen based on theoretical relevance and prior literature linking demographic and belief-related characteristics to COVID-19-related worry and preventive practices (16–18). Multicollinearity among predictor variables was assessed using the Variance Inflation Factor (VIF). The VIF values were examined for all included variables, and variables not exceeding the commonly accepted VIF threshold of 5 were selected (19).

To address potential multicollinearity and for clarity of interpretation among the two belief indices, two distinct models for both multivariate linear and logistic regression analyses were tested with only one of the belief indices used among the various features at a time. Including both indices as predictors in the same regression model, given their high correlation (*r* = 0.63) could inflate the standard errors of the estimated coefficients and lead to unstable estimates.

Finally, differences between Low vs. High worry groups and those who adhered vs. did not adhere to preventive measures were calculated using t-test for continuous variables and chi-square test for categorical variables. For all statistical analyses, including multivariate regression, chi-square tests, and t-tests, missing data were addressed by excluding observations with missing values for any of the key variables under consideration (i.e., complete case analyses).

Analyses were conducted using Python programming language Version 3.11.5, built-in Python features were used for calculating the descriptive statistics, pingouin library (20) was used for calculating Cronbach’s alpha, VIF was calculated using the statsmodel library (21), and scikit-learn library (22) was incorporated for both the multivariate and logistic regression models.

## Results

The sample consisted of 522 participants, who averaged 71.7 years (SD: 8.7), were evenly split between 70 years old or younger, were more likely to be women (73%) and African American (74%). The vast majority (90%) identified as non-Hispanic. Regarding residential distribution, 57% were from rural areas, while 43% resided in urban/suburban settings. In terms of education, most participants (66%) had 12 years or fewer of formal schooling, with the mean years of education being 12.1 (SD: 3.5). The majority of the participants were vaccinated (88%), had never tested positive for COVID-19 (64%), adhered to social distancing guidelines (73%), and used masks (57%). Most participants felt that the authorities were acting in their best interests (71%) and trusted the different sources of information available (81%; Table 1). Participant characteristics by level of post-COVID-19 worry and preventive behaviors are presented in Supplementary Tables 2, 3, respectively. The ‘Low Worry’ group accounted for 51%, while the ‘High Worry’ group accounted for 49%, suggesting that a slight majority of participants had less anxiety related to the pandemic. Ethnicity, vaccination status, locale distribution and post-COVID-19 worry had a significant influence on both social distancing and mask-wearing practices.

**Table 1:** Participant demographic and COVID-19 related characteristics.

Participant characteristics	Statistic
Sample size, N^a^	522
Age (years)	
Mean [SD]	71.7 (8.7)
>70	257 (50.1%)
≤70	256 (49.9%)
Sex, n (%)	
Female	358 (73.2%)
Male	131 (26.8%)
Race/ethnicity, n (%)	
White	130 (26.3%)
Black/African American	365 (73.7%)
Hispanic ethnicity, n (%)	
Yes	49 (9.7%)
No	455 (90.3%)
Education (years), mean [SD]	12.1 (3.5)
Years of Education ≤12	327 (65.9%)
Years of Education >12	169 (34.1%)
Urbanicity, n (%)	
Rural	270 (56.8%)
Urban/suburban	205 (43.2%)
COVID-19 Vaccination Status	
Vaccinated: Yes	394 (88.3%)
Vaccinated: No	52 (11.7%)
Tested positive for COVID-19: Yes	160 (35.8%)
Tested positive for COVID-19: No	287 (64.2%)
COVID-19 Preventive Measure	
Practiced Social Distancing	329 (74.4%)
Practiced Mask-Wearing	250 (56.4%)
COVID-19 belief Indices	
Best Interest Index, mean [SD]^b^	1.8 (0.6)
Best Interest: Agree^b^	251 (71.5%)
Best Interest: Disagree^b^	100 (28.5%)
Believe Information Index, mean [SD]^c^	1.9 (0.6)
Believe Information: Agree^c^	243 (80.7%)
Believe Information: Disagree ^c^	58 (19.3%)
COVID-19 Worry Index	4.5 (2.9)
COVID-19 Worry: higher, n (%)^d^	170 (48.6%)
COVID-19 Worry: lower, n (%)^d^	180 (51.4%)

a Missing data: Age, *n* = 9, Sex, *n* = 33, Racial group, *n* = 9; Hispanic ethnicity, *n* = 18, Education, *n* = 26, Urbanicity, *n* = 47, Vaccinated, *n* = 75, Tested positive for COVID-19, *n* = 74, Median worry score, *n* = 172, Best Interest Index, *n* = 120, Believe Information Index, *n* = 103.

b Believe City, county, state, and federal government looking out for best interest.

c Believe information from Doctor, Government, Pharmacy, TV.

d Dichotomized at median worry score (low: ≤4.67, high: >4.67; worry score range: 0-least worry to 10-most worry).

The internal consistency reliability for each index was assessed and found to be robust: Best Interest Index (*α* = 0.95, 95% CI: 0.94–0.96), Believe Information Index (*α* = 0.89, 95% CI: 0.87–0.90), and COVID-19 Worry Index (*α* = 0.96, 95% CI: 0.95–0.97). These results collectively indicate that all three indices possess strong internal consistency, supporting their use as reliable measures in this study.

We found several factors correlated with post-COVID-19 worry including select sociodemographic characteristics, prior COVID-19 test status, and the two belief measures, with the latter assessed in separate multivariate linear regression models (Table 2). All reported associations are correlational due to the cross-sectional design and should not be interpreted as causal relationships. For Model 1, Higher age (*β* = −0.05; 95% CI: −0.07, −0.03), living in urban/suburban areas (*β* = −1.37, 95% CI: −1.78, −0.96) and “ever testing positive” for COVID-19 (*β* = −0.56, 95% CI: −0.93, −0.19) were negatively associated with post-COVID-19 worry while Hispanic vs. non-Hispanic ethnicity (*β* = 0.75; 95% CI: 0.06, 1.43) was positively associated. Higher scores on the Best Interest Index (*β* = 0.51, 95% CI: 0.24, 0.78) were positively associated with post-COVID-19 worry. Similarly, for Model 2, higher scores on the Believe Information Index (*β* = 1.27, 95% CI: 1.02, 1.53) and Black vs. White racial background (*β* = 0.59, 95% CI: 0.09, 1.09) were positively associated. Male vs. female sex (*β* = −0.68; 95% CI: −1.06, −0.31), urban/suburban vs. rural setting (*β* = −1.20, 95% CI: −1.56, −0.83), and higher education (*β* = −0.06, 95% CI: −0.11, −0.01) were negatively associated with post-COVID-19 worry. Both models identified statistically significant associations between these various factors and the post-COVID-19 Worry Index, with some overlapping variables and some unique to each model.

**Table 2:** Adjusted associations between participant characteristics and worry about COVID-19 in 2022–2024.

Participant characteristic	Model 1^a^	Model 2^a^
	Estimate (95% CI)	Estimate (95% CI)
Best Interest Index^b^	0.51 (0.24, 0.78)	N/A
Believe Information Index^c^	N/A	1.27 (1.02, 1.53)
Age (years)	−0.05 (−0.07, −0.03)	−0.02 (−0.04, 0.00)
Male (vs. Female)	−0.40 (−0.82, 0.01)	−0.68 (−1.06, −0.31)
Black (vs. White)	0.53 (−0.02, 1.07)	0.59 (0.09, 1.09)
Hispanic ethnicity (vs. non-Hispanic)	0.75 (0.06, 1.43)	0.17 (−0.45, 0.80)
Education (years)	−0.02 (−0.07, 0.04)	−0.06 (−0.11, −0.01)
Urban/suburban (vs. rural)	−1.37 (−1.78, −0.96)	−1.20 (−1.56, −0.83)
Vaccinated (vs. not vaccinated)	0.44 (−0.16, 1.05)	0.14 (−0.39, 0.66)
Ever tested positive for COVID-19 (vs. never tested positive)	−0.56 (−0.93, −0.19)	0.12 (−0.20, 0.45)

CI, Confidence Interval; N/A, excluded from model. *Independent variables: Participant characteristics, Dependent variable: Worry Index. Analyses were conducted on complete cases only; individuals with missing data on key variables were excluded.

a Multivariable linear regression.

b Believe information from Doctor, Government, Pharmacy, TV.

c Believe City, county, state, and federal government looking out for best interest.

In multivariate logistic regression models assessing correlates of social distancing (Table 3), males were 0.37 times less likely than women (AOR = 0.37, 95% CI: 0.18, 0.76) and Black participants were 6 times more likely than White participants (AOR = 5.97, 95% CI: 2.3, 15.54) to practice social distancing in Model 1. When the Believe Information Index was included (Model 2), males were less likely than females (AOR = 0.39, 95% CI: 0.20, 0.78) and Black vs. White participants (AOR = 6.17, 95% CI 2.41, 15.79) and Hispanic vs. non-Hispanic (AOR = 3.42, 95%CI: 1.08, 10.87) participants were more likely to practice social distancing. In logistic regression models assessing associations with mask-wearing (Table 4), Black participants were 9 times more likely than White participants, Hispanics 5 times more likely than non-Hispanics (Models 1–2), and those vaccinated almost 3 times more likely (AOR = 2.70, 95%CI: 1.07, 6.79) to report masking in public spaces (Model 1).

**Table 3:** Adjusted association between participant characteristics and practice of social distancing in 2022–2024.

Participant characteristic	Model 1^a^	Model 2^a^
	AOR (95% CI)	AOR (95% CI)
Best Interest Index^b^	1.3 (0.78, 2.17)	N/A
Believe Information Index^c^	N/A	1.05 (0.62, 1.79)
Age (years)	0.97 (0.93, 1.01)	0.98 (0.95, 1.02)
Male (vs. Female)	0.37 (0.18, 0.76)	0.39 (0.2, 0.78)
Black (vs. White)	5.97 (2.3, 15.54)	6.17 (2.41, 15.79)
Hispanic ethnicity (vs. non-Hispanic)	3.17 (1.0, 10.11)	3.42 (1.08, 10.87)
Education (years)	1.04 (0.94, 1.14)	1.02 (0.93, 1.13)
Urban/suburban (vs. rural)	1.07 (0.46, 2.5)	1.23 (0.54, 2.82)
Vaccinated (vs. not vaccinated)	1.78 (0.65, 4.91)	2.23 (0.89, 5.6)
Ever tested positive for COVID-19 (vs. never tested positive)	1.38 (0.69, 2.79)	1.43 (0.74, 2.78)
COVID-19 worry index	1.06 (0.94, 1.19)	1.08 (0.96, 1.21)

AOR, Adjusted Odds Ratio; CI, Confidence Interval; N/A, excluded from model. Analyses were conducted on complete cases only; individuals with missing data on key variables were excluded.

a Multivariable logistic regression model.

b Believe information from Doctor, Government, Pharmacy, TV.

c Believe City, county, state, and federal government looking out for best interest.

**Table 4:** Adjusted associations between participant characteristics and masking in public settings in 2022–2024.

Participant characteristic	Model 1^a^	Model 2^a^
	AOR (95% CI)	AOR (95% CI)
Best Interest Index^b^	1.15 (0.77, 1.72)	N/A
Believe Information Index^c^	N/A	0.99 (0.64, 1.54)
Age (years)	1.0 (0.96, 1.03)	1.0 (0.97, 1.03)
Male (vs. Female)	0.63 (0.33, 1.19)	0.63 (0.34, 1.17)
Black (vs. White)	9.29 (3.73, 23.14)	9.59 (3.86, 23.78)
Hispanic ethnicity (vs. non-Hispanic)	4.84 (1.65, 14.17)	5.2 (1.78, 15.21)
Education (years)	0.99 (0.91, 1.07)	0.99 (0.92, 1.08)
Urban/suburban (vs. rural)	1.17 (0.62, 2.2)	1.15 (0.62, 2.13)
Vaccinated (vs. not vaccinated)	2.7 (1.07, 6.79)	2.27 (0.95, 5.42)
Ever tested positive for COVID-19 (vs. never tested positive)	1.21 (0.69, 2.13)	1.08 (0.63, 1.86)
COVID-19 worry index	1.02 (0.93, 1.12)	1.02 (0.93, 1.12)

AOR, Adjusted Odds Ratio; CI, Confidence Interval; N/A, excluded from model. Analyses were conducted on complete cases only; individuals with missing data on key variables were excluded.

a Multivariable logistic regression model.

b Believe information from Doctor, Government, Pharmacy, TV.

c Believe City, county, state, and federal government looking out for best interest.

## Discussion

In a diverse sample of community-dwelling older adults, sociodemographic characteristics (age, sex, race/ethnicity, education, place of residence) and COVID-19–related factors (beliefs, vaccine status, testing status, trust in authorities and information sources) were significantly associated with level of concern about COVID-19 and adherence to preventive practices such as social distancing and masking. Notably, Black racial identity was the most significant correlate of adherence to both social distancing and masking, highlighting key individual characteristics associated with post-COVID-19 worry and preventive practices following the peak of the COVID-19 pandemic, after vaccinations became available.

Most surveyed participants expressed positive beliefs about government intentions and found information from authorities (including government officials, doctors, drug companies, and media) to be trustworthy. Increased exposure to and trust in official information sources was positively correlated with higher post-COVID-19 worry, likely because those who trust official sources may be more inclined to absorb and internalize the data they encountered (23).

This finding aligns with behavioral science models such as the Health Belief Model (HBM), which posits that perceived risk and benefits of action are key drivers of preventive health behaviors (24). In our study, heightened trust in official sources may have increased perceived risk, thereby motivating adherence to preventive measures in certain demographics. Factors such as lower income, limited resources, and greater exposure to COVID-19 may have also heightened perceived susceptibility and severity, thereby leading to increased adoption of preventive behaviors. Additionally, the Theory of Planned Behavior (25) emphasizes the influence of attitudes, subjective norms, and perceived behavioral control on health-related decisions. In our population, subjective norms such as community expectations and leadership likely played a significant role, particularly in the Black community, where targeted education and advocacy by trusted leaders may have reinforced protective behaviors. These patterns reflect the influence of local social determinants of health, including socioeconomic status, geographic isolation, and systemic inequities in access to healthcare and information.

In our population, perceived susceptibility and severity may have been heightened among those with lower income, limited resources, and greater exposure to COVID-19, leading to increased preventive behaviors. Subjective norms such as community expectations and leadership likely played a significant role, particularly in the Black community, where targeted education and advocacy by trusted leaders may have reinforced protective behaviors. Additionally, local cultural norms and health literacy levels may have influenced how information was received and acted upon. In the rural setting, community-based organizations and faith-based institutions may have served as trusted sources of information, thereby shaping both risk perception and behavioral responses.

Higher post-COVID-19 related concerns observed in women may be due to disproportionate impacts of caregiving, child-bearing and economic vulnerabilities (26). Elevated post-COVID-19 worry among Black older adults and rural inhabitants may be due to their overrepresentation in lower income groups, preexisting vulnerabilities, and limited access to resources (27, 28). These patterns reflect the influence of local social determinants of health, including socioeconomic status, geographic isolation, and systemic inequities in access to healthcare and information. In the context of our surveyed sample, particularly in “The Glades,” these factors are compounded by historical underinvestment in community resources and health infrastructure, which may have heightened perceived risk and motivated protective behaviors.

Conversely, age and years of education were inversely related to post-COVID-19 concerns. A study examining age differences in COVID-19 risk perceptions and mental health found that older age was associated with lower risk perceptions for contracting COVID-19 possibly due to higher vaccination rates (29), a more positive outlook in later life and better emotion regulation (30).

Relationship between years of education and post-COVID-19 worry may be influenced by field of study, exposure to COVID-19 patients, and individual circumstances (31, 32). Another study indicated that higher education was linked to lower risk perception and reduced anxiety, likely due to greater access to information, higher health literacy, and better coping mechanisms (33). However, the literature is not entirely uniform. Some studies have found only small or non-significant differences in worry by education level, and, in certain contexts, higher education has been associated with increased worry, possibly due to a more nuanced understanding of risks (34, 35). For instance, in specific phases of the pandemic, highly educated men reported more worries than their less-educated counterparts (34). Overall, most evidence supports the protective role of education against excessive worry, although the relationship may depend on context, age, and gender (34, 35). Notably, the interplay between education and health literacy is particularly relevant in our study population, where cultural norms and community-based education efforts may have shaped both the interpretation of risk and the adoption of preventive behaviors.

We observed that women and Black older adults were more likely to practice social distancing. Survey data from eight countries revealed significantly higher adherence with public health and social distancing guidelines among women (78%) compared to men (72%) (36). Compared to sex, racial identities showed a much greater contrast when it came to social distancing. In our study, Black participants had a much higher social distancing rate of 82% when compared to the White participants (55%), which is in stark contrast to other studies. A nationally representative survey of 604 Black participants found much lower adherence to four key COVID-19 public health recommendations with social distancing among Black individuals being one of them (37). Similarly, another study conducted in Michigan revealed that 42% of African American individuals adhered to social distancing measures (38).

To further contextualize these findings, it is important to note the unique community dynamics in “The Glades.” According to local health planning documents, community-based organizations, faith-based groups, and local health departments in Glades County have historically played a central role in disseminating health information and promoting preventive behaviors (39). During the pandemic, these groups intensified outreach through church announcements, community meetings, and partnerships with trusted messengers, such as pastors and civic leaders. Additionally, the Glades community has faced longstanding disparities in healthcare access and infrastructure, which may have heightened awareness of vulnerability and motivated collective protective behaviors (39). These locally tailored, trust-based interventions likely contributed to the observed high rates of social distancing among Black older adults in our sample. Such strategies could be capitalized upon in the event of future public health crises.

Several factors were associated with higher likelihood of masking in public, including Black or Hispanic identity, and COVID-19 vaccine recipients. Similar results were seen in other studies done nationwide when comparing mask wearing among different ethnoracial groups. A study conducted at the University of Buffalo, found that in Erie County, Black women had the highest rates of mask adherence while overall mask adherence among Black individuals was 4 times that of White people in the county (40). In another study the average predicted probability of mask wearing was reported at 0.92 for Black respondents, compared to 0.84 for White respondents (41). Factors that lead to increased adherence to social distancing guidelines, could also be attributed to increased mask wearing in the African American survey responders (37, 42). On the other hand, increased mask wearing in COVID-19 vaccinated individuals may reflect high risk perception, consistent protective behavior, and trust in public health guidance (43, 44).

Our study has certain limitations to consider. The study’s cross-sectional design and absence of baseline data prior to widespread testing and vaccine availability make it challenging to understand long-term trends and changes in post-COVID-19 concerns and preventive practices. Differences in testing and reporting practices between states make comparisons across state lines challenging. The rapidly evolving pandemic situation likely influenced survey responses, adding another layer of complexity to the analysis. As the study used a cross-sectional design, all observed relationships are associative and cannot be taken as evidence of causation.

While several associations reached statistical significance, some effect sizes were small and may have limited practical or public health significance. For example, although higher age (Model 1) and education (Model 2) were statistically associated with lower post-COVID-19 worry, the absolute effect sizes (*β* coefficients) were modest. These findings should be interpreted with consideration of both statistical and clinical significance.

Another limitation of this study was the use of a convenience sample, with 522 participants recruited through word-of-mouth, flyers, and recruitment events across the different residential settings. This non-probability sampling approach may have introduced selection bias, as individuals who chose to participate could differ systematically from those who did not. Specifically, the sample may overrepresent individuals who were more socially engaged, more health-conscious, or more concerned about COVID-19, as these individuals may be more likely to notice recruitment materials or attend recruitment events. Consequently, the findings may not be fully generalizable to the broader population, particularly to those who are less socially active, less concerned about COVID-19, or less likely to participate in community events. This potential overrepresentation could influence the observed attitudes, behaviors, or experiences related to COVID-19 within the study, and should be considered when interpreting the results.

While the study faced some missing data for certain key variables, this was addressed by dropping missing values and conducting complete case analyses. This approach may have biased study findings if missingness was not completely at random.

As the survey was administered exclusively in English, this may have introduced selection bias. While English is widely spoken in South Florida, this language restriction may have excluded non-native English speakers, particularly those who are more comfortable communicating in Spanish, Haitian Creole, or other languages prevalent in the region. As a result, the sample may not fully represent the linguistic and cultural diversity of the broader South Florida population, potentially underrepresenting the experiences and perspectives of non-English-speaking residents and thereby reducing the inclusivity and generalizability of the study findings.

## Conclusion

The COVID-19 pandemic has had a significant global impact, revealing the complex web of social, economic, and cultural factors that influence disease control and prevention. Our research showed that sociodemographic characteristics—particularly race/ethnicity, sex, locale, and education—as well as COVID-19–related beliefs and vaccination status, were significantly associated with both post-COVID-19 worry and adherence to preventive behaviors such as social distancing and mask-wearing. Notably, Black and Hispanic participants, women, and those who trusted official information sources or were vaccinated, demonstrated higher rates of preventive practices. These findings highlight the importance of culturally tailored, trust-based public health strategies, especially in diverse and underserved communities. Our results suggest that future public health interventions should prioritize building trust in official information, leveraging community-based organizations, and addressing the unique needs of specific demographic groups to enhance the effectiveness of preventive measures. For instance, engaging faith-based and local leaders in outreach efforts may be particularly impactful in rural or minority communities. Our findings should be interpreted considering several limitations, including the cross-sectional and correlational nature of the study, use of a convenience sample, and English-only survey administration, which may limit generalizability.

In conclusion, our study underscores the need for nuanced, context-specific public health strategies that recognize the interplay of sociodemographic factors, trust, and community dynamics in shaping preventive behaviors. Addressing these factors is vital for improving health equity and resilience in the face of future public health emergencies, particularly in diverse regions such as South Florida.

## Data Availability

The raw data supporting the conclusions of this article will be made available by the authors, without undue reservation.
