# Supplementary material for: Correlates of post-COVID-19 pandemic worry and preventive practices in older adults in Florida
Source: Front Public Health. 2025 Jul 9;13:1608352. doi: 10.3389/fpubh.2025.1608352 (PMC12283615; doi:10.3389/fpubh.2025.1608352)
Supplement: Supplementary file 1 [file Table_1.docx]

Supplemental Table 1. Covid survey

**Participant ID: ___________________________________ Visit Date: ___________**

1. In the past week, how much, if anything have you heard or read about COVID-19?

____Nothing

____A little

____A moderate amount

____A lot

2. In the past week, did you see more or less news than you wanted to see about COVID-19?

____Much more

____More

____About the right amount

____Less

____Much less

3. Have you ever tested positive for COVID-19?

___Yes ___ No

3a. If Yes, how many time have you tested positive? _______

3b. If Yes, have you taken any of the antiviral medications for COVID-19?

___Yes ___ No

4. Have you received the vaccine for COVID-19

___Yes ___ No

5. Do you practice social distancing when in public settings?

___Yes ___ No

5a. If Yes, do others shame you or make comments about you practicing social distancing?

___Yes ___ No

5b. If No, does it bother you if others practice social distancing?

___Yes ___ No

6. Do you wear a mask in public settings?

___Yes ___ No

6a. If Yes, do others shame you or make comments about you wearing a mask?

___Yes ___ No

6b. If No, does it bother you if others wear masks?

___Yes ___ No

**The following questions examine how COVID-19 may have impacted your life. Please rate how much you agree with the following statements.**

| **How much do you agree with the following….** | **Strongly**  **Disagree** | **Disagree** | **Agree** | **Strongly**  **Agree** |
| --- | --- | --- | --- | --- |
| I believe what government officials tell me about COVID-19 |  |  |  |  |
| I believe what doctors tell me about COVID-19 |  |  |  |  |
| I believe what drug companies tell me about COVID-19 |  |  |  |  |
| I believe what TV news reporters tell me about COVID-19 |  |  |  |  |
| The city government is looking out for my best interest |  |  |  |  |
| The county government is looking out for my best interest |  |  |  |  |
| The state government is looking out for my best interest |  |  |  |  |
| The federal government is looking out for my best interest |  |  |  |  |

**Using the scale below from 0 to 10, where 10 means worrying about this all the time and 0 means not thinking about it at all, how would you rate your feelings and thoughts about the following statements?**


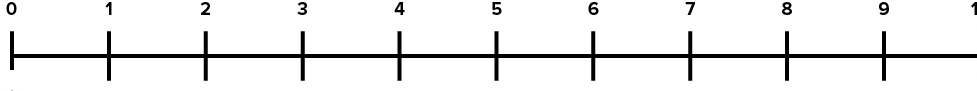


**Never think about it Think about 50% of the time Always thinking about it**

| **On a scale of 0 to 10, how much do you worry about the following.** | **Rating**  **0-10** |
| --- | --- |
| I worry about getting sick with a new case of COVID-19 |  |
| I worry about the next strain or variant of COVID-19 |  |
| I worry about the long-term effects of COVID-19 on my physical health |  |
| I worry about the long-term effects of COVID-19 on my mental health |  |
| I worry about the long-term effects of COVID-19 on my brain |  |
| I worry about the potential unknown long-term effects of the COVID-19 vaccine |  |
| I worry about long-term economic effects of COVID-19 on my community |  |
| I worry about long-term social isolation effects of COVID-19 on my community |  |
| I worry about long-term government policy effects of COVID-19 on my community |  |
| I worry that things will never return to the way they were before COVID-19 |  |
| I worry that we will not be prepared for the next virus that affects my community |  |
| I worry that all the personal safety measures that I have done may be for nothing |  |

Supplemental Table 2. Participant characteristics by level of worry about COVID-19 in 2023-2024

| **Participant characteristic^s^** | **Low worry^b^** | **High worry^b^** | ***P*^c^** |
| --- | --- | --- | --- |
| Sample size, N | 180(51.4%) | 170(48.6%) | N/A |
| Age (years) |  |  |  |
| Mean (SD) | 71.8(8.6) | 70.4(8.2) | 0.14 |
| >70 | 89(53.9%) | 76(46.1%) | 0.38 |
| ≤70 | 89(48.6%) | 94(51.4%) |  |
| Sex, n (%) |  |  |  |
| Female | 130(51.4%) | 123(48.6%) | 0.94 |
| Male | 38(50.0%) | 38(50.0%) |  |
| Race/ethnicity, n (%) |  |  |  |
| White | 56(65.9%) | 29(34.1%) | <0.0001 |
| Black/African American | 117(46.8%) | 133(53.2%) |  |
| Hispanic ethnicity, n (%) |  |  |  |
| Yes | 18(51.4%) | 17(48.6%) | 1.00 |
| No | 159(51.3%) | 151(48.7%) |  |
| Education (years), mean (SD) | 12.5(3.6) | 12.0(3.1) | 0.12 |
| Years of Education≤12 | 105(47.1%) | 118(52.9%) | 0.03 |
| Years of Education>12 | 70(59.8%) | 47(40.2%) |  |
| Urbanicity, n (%) |  |  |  |
| Rural | 82(43.4%) | 107(56.6%) | <0.0001 |
| Urban/suburban | 97(61.4%) | 61(38.6%) |  |
| COVID-19 Vaccination Status |  |  |  |
| Vaccinated: Yes | 152(49.0%) | 158(51.0%) | 0.04 |
| Vaccinated: No | 26(68.4%) | 12(31.6%) |  |
| Tested positive for COVID-19: Yes | 68(54.8%) | 56(45.2%) | 0.36 |
| Tested positive for COVID-19: No | 110(49.1%) | 114(50.9%) |  |
| COVID-19 belief Indices |  |  |  |
| Best Interest Index, mean (SD)^d^ | 1.7(0.6) | 1.8(0.7) | 0.40 |
| Best Interest: Agree ^d^ | 92(48.2%) | 99(51.8%) | 0.54 |
| Best Interest: Disagree ^d^ | 43(53.1%) | 38(46.9%) |  |
| Believe Information Index, mean (SD)^e^ | 1.8(0.6) | 2.0(0.6) | <0.0001 |
| Believe Information: Agree ^e^ | 85(45.5%) | 102(54.5%) | 0.02 |
| Believe Information: Disagree ^e^ | 32(65.3%) | 17(34.7%) |  |
| ^a^ Missing data: Age, n=9, Gender, n=33, Racial group, n=9; Hispanic ethnicity, n=18, Education, n=26, Urbanicity, n=47, Vaccinated, n=75, Tested positive for COVID-19, n=74, Median worry score, n=172, Best Interest Index, n=120, Believe Information Index, n=103  ^b^ Dichotomized at median worry score (low: ≤4.67, high: >4.67) (worry score range: 0-least worry to 10-most worry)  ^c^ t-test or chi-square test  ^d^ Believe City, county, state, and federal government looking out for best interest  ^e^ Believe information from Doctor, Government, Pharmacy, TV | | | |

Supplemental Table 3. Participant characteristics by social distancing and masking in public settings in 2023-2024

| **Participant characteristics** | **Social distancing in public settings** | | ***P*^b^** | **Mask in public settings** | | ***P*^b^** |
| --- | --- | --- | --- | --- | --- | --- |
|  | **Yes** | **No** |  | **Yes** | **No** |  |
| Sample size, N | 329(73.9%) | 116(26.1%) | N/A | 250(56.4%) | 193(43.6%) | N/A |
| Age (years), mean (SD) | 71.31 | 72.69 | 0.16 | 71.73 | 71.55 | 0.84 |
| Sex, n (%) |  |  |  |  |  |  |
| Female | 246(77.1%) | 73(22.9%) | 0.04 | 187(58.8%) | 131(41.2%) | 0.13 |
| Male | 66(66.0%) | 34(34.0%) |  | 49(49.5%) | 50(50.5%) |  |
| Race/ethnicity, n (%) |  |  |  |  |  |  |
| White | 59(54.6%) | 49(45.4%) | <0.0001 | 28(25.7%) | 81(74.3%) | <0.0001 |
| Black/African American | 257(81.6%) | 58(18.4%) |  | 209(66.8%) | 104(33.2%) |  |
| Hispanic ethnicity, n (%) |  |  |  |  |  |  |
| Yes | 32(71.1%) | 13(28.9%) | 0.76 | 23(50.0%) | 23(50.0%) | 0.43 |
| No | 292(74.5%) | 100(25.5%) |  | 223(57.3%) | 166(42.7%) |  |
| Education (years), mean (SD) | 11.96 | 12.02 | 0.88 | 11.63 | 12.35 | 0.04 |
| Urbanicity, n (%) |  |  |  |  |  |  |
| Rural | 187(78.6%) | 51(21.4%) | 0.03 | 150(63.3%) | 87(36.7%) | <0.0001 |
| Urban/suburban | 139(68.8%) | 63(31.2%) |  | 97(48.3%) | 104(51.7%) |  |
| COVID-19 Vaccination Status |  |  |  |  |  |  |
| Vaccinated: Yes | 297(76.0%) | 94(24.0%) | 0.01 | 228(58.8%) | 160(41.2%) | 0.01 |
| Vaccinated: No | 29(56.9%) | 22(43.1%) |  | 20(38.5%) | 32(61.5%) |  |
| Previously tested positive for COVID-19 | 111(69.8%) | 48(30.2%) | 0.18 | 84(53.2%) | 74(46.8%) | 0.34 |
| COVID-19 worry index | 4.79 | 3.64 | <0.0001 | 4.86 | 3.97 | 0.01 |
| COVID-19 worry: higher, n (%) ^c^ | 137(81.1%) | 32(18.9%) | 0.05 | 104(63.0%) | 61(37.0%) | 0.03 |
| COVID-19 worry: lower, n (%)^c^ | 128(71.5%) | 51(28.5%) |  | 91(50.8%) | 88(49.2%) |  |
| Best Interest Index, mean (SD)^d^ | 1.81 | 1.69 | 0.10 | 1.8 | 1.77 | 0.65 |
| Best Interest: Agree, n (%)^d^ | 192(76.8%) | 58(23.2%) | 0.17 | 139(56.3%) | 108(43.7%) | 1.00 |
| Best Interest: Disagree, n (%)^d^ | 69(69.0%) | 31(31.0%) |  | 56(56.6%) | 43(43.4%) |  |
| Believe Information Index, mean (SD)^e^ | 1.90 | 1.79 | 0.14 | 1.89 | 1.84 | 0.43 |
| Believe Information: Agree, n (%)^e^ | 184(76.0%) | 58(24.0%) | 0.64 | 141(59.2%) | 97(40.8%) | 0.86 |
| Believe Information: Disagree, n (%)^e^ | 41(71.9%) | 16(28.1%) |  | 33(56.9%) | 25(43.1%) |  |
| ^a^ Missing data: Age, n=9, Gender, n=33, Racial group, n=9; Hispanic ethnicity, n=18, Education, n=26, Urbanicity, n=47, Vaccinated, n=75, Tested positive for COVID-19, n=74, Median worry score, n=172, Best Interest Index, n=120, Believe Information Index, n=103  ^b^ t-test or chi-square test  ^c^ Dichotomized at median worry score (low: ≤4.67, high: >4.67) (worry score range: 0-least worry to 10-most worry)  ^d^ Believe City, county, state, and federal government looking out for best interest  ^e^ Believe information from Doctor, Government, Pharmacy, TV | | | | | | |

**Worry index- scored on a scale of 0-10, with 0 being the least and 10 being the highest amount of worry*
